# More animals than markers: a study into the application of the single step T-BLUP model in large-scale multi-trait Australian Angus beef cattle genetic evaluation

**DOI:** 10.1186/s12711-019-0499-x

**Published:** 2019-10-16

**Authors:** Vinzent Boerner, David J. Johnston

**Affiliations:** Animal Genetics and Breeding Unit (AGBU), Armidale, Australia

## Abstract

Multi-trait single step genetic evaluation is increasingly facing the situation of having more individuals with genotypes than markers within each genotype. This creates a situation where the genomic relationship matrix ($\mathbf{G }$) is not of full rank and its inversion is algebraically impossible. Recently, the SS-T-BLUP method was proposed as a modified version of the single step equations, providing an elegant way to circumvent the inversion of the $\mathbf{G }$ and therefore accommodate the situation described. SS-T-BLUP uses the Woodbury matrix identity, thus it requires an add-on matrix, which is usually the covariance matrix of the residual polygenic effet. In this paper, we examine the application of SS-T-BLUP to a large-scale multi-trait Australian Angus beef cattle dataset using the full BREEDPLAN single step genetic evaluation model and compare the results to the application of two different methods of using $\mathbf{G }$ in a single step model. Results clearly show that SS-T-BLUP outperforms other single step formulations in terms of computational speed and avoids approximation of the inverse of $\mathbf{G }$.

## Background

Within the last decade, genotyping thousands of individuals with single nucleotide polymorphism (SNP) chips has become common practice in breeding programs of many species of economic relevance. However, due to cost effectiveness these individuals are being genotyped with low- to medium-density SNP chips, with usually not more than 50,000 markers.

To date, genetic evaluation systems accommodate SNP genotypes via the so-called single step model, in which most often markers are used to pre-calculate a relationship matrix, which subsequently augments the usual pedigree derived relationship matrix into a so-called $\mathbf{H }$ matrix  (SS-H-BLUP) [1]. With the mixed model equations (MME) requiring the inverse of this matrix, and assuming that $\mathbf{G }$ is actually algebraically invertible, increasing numbers of genotyped individuals have imposed a large computational burden on genetic evaluation systems. To circumvent this problem an approximation of the inverse of $\mathbf{G }$ was proposed, but the effect of this approximation on estimated breeding values (EBV) is dataset-dependent and must therefore be empirically determined for every single application [2].

However, the situation described above of having more genotyped individuals than markers has led to a situation where $\mathbf{G }$ is not of full rank and therefore algebraically no longer invertible. An alternative solution is to not use $\mathbf{G }$ and move to a model which incorporates the markers directly (SS-SNP-BLUP). While SS-SNP-BLUP is generally equivalent to SS-H-BLUP, and some formulations such as [3] offer huge model flexibility, many of its final implementations suffer from convergence problems with regard to iterative solving [3] or demanding pre-conditioner computation [4]. However, recently an elegant intermediate model has been formulated, which may be seen as a mix of SS-H-BLUP and SS-SNP-BLUP and is called SS-T-BLUP [5, 6]. SS-T-BLUP does not need $\mathbf{G }$ or its inverse and fits the marker indirectly. As it also fits $\mathbf{G }$ indirectly, it is generally algebraically equivalent to SS-H-BLUP. Thus, it provides EBV at the individual level, which can be readily transformed into SNP solutions but avoids the complex co-variance structure of SS-SNP-BLUP [3, 5, 7].

In this paper, we will examine the computational advantage of SS-T-BLUP for a large-scale multi-trait BREEDPLAN single step genetic evaluation of Australian Angus beef cattle. We will compare the results to those obtained by using an ordinary SS-H-BLUP approach.

## Methods

### Model

In the following, three equivalent representations of the inverse of the $\mathbf{H }$ matrix are derived which differ in their computational demand before and while solving the MME. Many of the formulas have been derived elsewhere [1, 5, 6, 8–11], but for convenience they are presented below.

The $\mathbf{H }$ matrix required for SS-H-BLUP can be written as:

1 $$\begin{aligned} \begin{array}{c|c} \mathbf{A }_{1,1}-\mathbf{A }_{1,2}\mathbf{A }_{2,2}^{-1}\mathbf{A }_{2,1}+\mathbf{A }_{1,2}\mathbf{A }_{2,2}^{-1}\mathbf{G }_\mathbf{w }(\mathbf{A }_{1,2}\mathbf{A }_{2,2}^{-1})^\prime & (\mathbf{A }_{1,2}\mathbf{A }_{2,2}^{-1})\mathbf{G }_\mathbf{w }\\ \hline \mathbf{G }_\mathbf{w }(\mathbf{A }_{1,2}\mathbf{A }_{2,2}^{-1})^\prime & \mathbf{G }_\mathbf{w }\\ \end{array} \end{aligned}$$

where $\mathbf{A }$ is the pedigree-based numerator relationship matrix, $\mathbf{A }_{1,1}$ denotes a diagonal block of $\mathbf{A }$ related to the set of $m_n$ non-genotyped individuals, $\mathbf{A }_{2,2}$ denotes a diagonal block of $\mathbf{A }$ related to the set of $m_g$ genotyped individuals, and $\mathbf{A }_{1,2}$ and $\mathbf{A }_{2,1}$ denote off-diagonal blocks of $\mathbf{A }$ located between the non-genotyped and genotyped individuals. $\mathbf{G }_{\mathbf{w }}$ is a genomic relationship matrix of dimension $m_{g} \times m_{g}$ which is constructed by $\mathbf{G }_{\mathbf{w }}$ = $\gamma$ $\mathbf{M }$ $\mathbf{D }$ $\mathbf{M}^\prime+$ $\lambda$ $\mathbf{C }$, where $\mathbf{M }$ is a centred and scaled matrix of marker genotypes of dimension $m_g \times m_m$, $\mathbf{D }$ is an arbitrary but symmetric and positive definite matrix of dimension $m_m \times m_m$, $\mathbf{C }$ is an arbitrary but symmetric and positive definite matrix of dimension $m_g \times m_g$, and $\gamma$ and $\lambda$ are arbitrary non-zero weights. Note that in applications where all markers are weighted equally and the co-variance between markers is set to zero, $\mathbf{D }$ reduces to an identity matrix if $\mathbf{M }$ is centred and scaled. Furthermore, $\mathbf{C }$ may be a diagonal matrix of random noise which ensures invertibility of $\mathbf{M }$ $\mathbf{D }$ $\mathbf{M }^\prime$, and $\lambda$ and $\gamma$ are set to 1. Or $\mathbf{C }$ = $\mathbf{A }_{2,2}$, $0<\lambda <1$, $\gamma =1-\lambda$, where $\lambda$ is interpreted as the proportion of the total additive genetic variance not explained by markers [8].

$\mathbf{H }^{-1}$ can be written as:

2 $$\begin{aligned} \left( \begin{array}{c|c} \mathbf{A }^{1,1}& \mathbf{A }^{1,2} \\ \hline \mathbf{A }^{2,1} & \mathbf{A }^{2,2}\\ \end{array} \right) + \left( \begin{array}{c|c} 0 & 0 \\ \hline 0 & \mathbf{G }_\mathbf{w }^{-1}- \mathbf{A }_{2,2}^{-1} \end{array} \right) \end{aligned},$$

or, replacing $\mathbf{A }_{2,2}^{-1}$ by ($\mathbf{A }^{2,2}-\mathbf{A }^{2,1}(\mathbf{A }^{1,1})^{-1}\mathbf{A }^{1,2}$), as $\widetilde{\mathbf{H }}^{-1}$

3 $$\begin{aligned} \left( \begin{array}{c|c} \mathbf{A }^{1,1}& \mathbf{A }^{1,2} \\ \hline \mathbf{A }^{2,1} & \mathbf{A }^{2,2}\\ \end{array} \right) + \left( \begin{array}{c|c} 0 & 0 \\ \hline 0 & \mathbf{G }_\mathbf{w }^{-1}- (\mathbf{A }^{2,2}-\mathbf{A }^{2,1}(\mathbf{A }^{1,1})^{-1}\mathbf{A }^{2,1}) \end{array} \right) , \end{aligned}$$

where $\mathbf{A }^{:,:}$ is a respective block of the inverse of $\mathbf{A }$.

Replacing $\mathbf{G }_{\mathbf{w }}$ with $\gamma$ $\mathbf{M }$ $\mathbf{D }$ $\mathbf{M }^\prime$ + $\lambda$ $\mathbf{C }$ in Eq. 1 and inverting the resulting matrix yields:

4 $$\begin{aligned} \begin{array}{l} \left( \begin{array}{c|c} \mathbf{A }^{1,1} & \mathbf{A }^{1,2} \\ \hline \mathbf{A }^{2,1} & \mathbf{A }^{2,2}\\ \end{array} \right) - \left( \begin{array}{c|c} 0 & 0 \\ \hline 0 & \mathbf{A }_{2,2}^{-1}\\ \end{array} \right) \\ +\left( \begin{array}{c|c} 0 & 0 \\ \hline 0 & \lambda ^{-1}\mathbf{C }^{-1}-\lambda ^{-1}\mathbf{C }^{-1}\mathbf{M }(\gamma ^{-1}\mathbf{D }^{-1}+\mathbf{M }'(\lambda ^{-1}\mathbf{C }^{-1})\mathbf{M })^{-1}M'\mathbf{C }^{-1}\lambda ^{-1} \end{array} \right) , \end{array} \end{aligned}$$

where

5 $$\begin{aligned} (\gamma \mathbf{M }\mathbf{D }\mathbf{M}^\prime+ \lambda \mathbf{C })^{-1}=\lambda ^{-1}\mathbf{C }^{-1}-\lambda ^{-1}\mathbf{C }^{-1}\mathbf{M }(\gamma ^{-1}\mathbf{D }^{-1}+\mathbf{M }^\prime(\lambda ^{-1}\mathbf{C }^{-1})\mathbf{M })^{-1}\mathbf{M }^\prime\mathbf{C }^{-1}\lambda ^{-1} \end{aligned}$$

according to the Woodbury matrix identity.

Assuming that $\mathbf{C }^{-1}$ = $\mathbf{A }_{2,2}^{-1}$, Eq. 4 simplifies to:

6 $$\begin{aligned} \begin{array}{ll} \left( \begin{array}{c|c} \mathbf{A }^{1,1} & \mathbf{A }^{1,2} \\ \hline \mathbf{A }^{2,1} & \mathbf{A }^{2,2}\\ \end{array} \right) + \left( \begin{array}{c|c} 0 & 0 \\ \hline 0 & (\lambda ^{-1}-1)\mathbf{A }_{2,2}^{-1}\\ \end{array} \right) \\ -\left( \begin{array}{c|c} 0 & 0 \\ \hline 0 & \lambda ^{-1}\mathbf{A }_{2,2}^{-1}\mathbf{M }(\gamma ^{-1}\mathbf{D }^{-1}+\mathbf{M }^\prime(\lambda ^{-1}\mathbf{A }_{2,2}^{-1})\mathbf{M })^{-1}\mathbf{M }^\prime\mathbf{A }_{2,2}^{-1}\lambda ^{-1} \end{array} \right) . \end{array} \end{aligned}$$

Setting $\mathbf{M }^{\dagger }$ = $\lambda ^{-1}$ $\mathbf{A }_{2,2}^{-1}$ $\mathbf{M }$ reduces Eq. 6 to:

7 $$\begin{aligned} \begin{array}{l} \left( \begin{array}{c|c} \mathbf{A }^{1,1} & \mathbf{A }^{1,2} \\ \hline \mathbf{A }^{2,1} & \mathbf{A }^{2,2}\\ \end{array} \right) + \left( \begin{array}{c|c} 0 & 0 \\ \hline 0 & (\lambda ^{-1}-1)\mathbf{A }_{2,2}^{-1}\\ \end{array} \right) -\left( \begin{array}{c|c} 0 & 0 \\ \hline 0 & \mathbf{M }^{\dagger }(\gamma ^{-1}\mathbf{D }^{-1}+\mathbf{M }^\prime\mathbf{M }^{\dagger })^{-1}\mathbf{M }^{\dagger ^\prime} \end{array} \right) . \end{array} \end{aligned}$$

Furthermore, defining $\mathbf{K }_{\mathbf{u }}$ as the upper Cholesky factor of matrix ($\gamma ^{-1}$ $\mathbf{D }^{-1}$ + $\mathbf{M }^{^\prime}$ $\mathbf{M }^{\dagger }$) simplifies Eq. 7 to:

8 $$\begin{aligned} \left( \begin{array}{c|c} \mathbf{A }^{1,1} & \mathbf{A }^{1,2} \\ \hline \mathbf{A }^{2,1} & \mathbf{A }^{2,2}\\ \end{array} \right) + \left( \begin{array}{c|c} 0 & 0 \\ \hline 0 & (\lambda ^{-1}-1)\mathbf{A }_{2,2}^{-1}\\ \end{array} \right) -\left( \begin{array}{c|c} 0 & 0 \\ \hline 0 &\ \mathbf{M }^{\dagger }(\mathbf{K }_u)^{-1}(\mathbf{K }_u^{^\prime})^{-1}\mathbf{M }^{\dagger ^\prime} \end{array} \right) \end{aligned},$$

which, when setting $\mathbf{M }^{*}$ = $\mathbf{M }^{\dagger }$($\mathbf{K }_{\mathbf{u }}$)$^{-1}$simplifies to

9 $$\begin{aligned} \left( \begin{array}{c|c} \mathbf{A }^{1,1} & \mathbf{A }^{1,2} \\ \hline \mathbf{A }^{2,1} & \mathbf{A }^{2,2}\\ \end{array} \right) + \left( \begin{array}{c|c} 0 & 0 \\ \hline 0 & (\lambda ^{-1}-1)\mathbf{A }_{2,2}^{-1}\\ \end{array} \right) -\left( \begin{array}{c|c} 0 & 0 \\ \hline 0 & \mathbf{M }^{*}\mathbf{M }^{*^\prime} \end{array} \right) \end{aligned}.$$

Following the derivation of $\widetilde{\mathbf{H }}^{-1}$, replacing $\mathbf{A }_{2,2}^{-1}$ in Eq. 9 by ($\mathbf{A }^{2,2}-\mathbf{A }^{2,1}(\mathbf{A }^{1,1})^{-1}\mathbf{A }^{1,2}$) yields matrix ${\varvec{\Psi }}^{-1}$:

10 $$\begin{aligned} \left( \begin{array}{c|c} \mathbf{A }^{1,1} & \mathbf{A }^{1,2} \\ \hline \mathbf{A }^{2,1} & \mathbf{A }^{2,2}\\ \end{array} \right) + \left( \begin{array}{c|c} 0 & 0 \\ \hline 0 & (\lambda ^{-1}-1)(\mathbf{A }^{2,2}-\mathbf{A }^{2,1}(\mathbf{A }^{1,1})^{-1}\mathbf{A }^{2,1})\\ \end{array} \right) -\left( \begin{array}{c|c} 0 & 0 \\ \hline 0 & \mathbf{M }^{*}\mathbf{M }^{*^\prime} \end{array} \right) . \end{aligned}$$

Given the matrices $\mathbf{H }^{-1}$, $\widetilde{\mathbf{H }}^{-1}$ and ${\varvec{\Psi }}^{-1}$, three different BLUP models can be defined, SS-H-BLUP, SS-$\widetilde{\text {H}}$-BLUP, and SS-T-BLUP, which differ solely in the formulation of the inverse of $\mathbf{H }$ used ($\mathbf{H }^{-1}$, $\widetilde{\mathbf{H }}^{-1}$ or ${\varvec{\Psi }}^{-1}$).

### Computational implications when solving iteratively

The differences between the three approaches regarding computational time spent on preparing necessary data and solving the MME iteratively can be reduced to a set of very specific operations unique to the respective representation of the inverse of $\mathbf{H }$. This also applies to the differences in memory requirements.

Assuming that $\mathbf{C }$ = $\mathbf{A }_{2,2}$, preparation of SS-H-BLUP requires to build $\mathbf{G }$, $\mathbf{A }_{2,2}$ and $\mathbf{G }_{\mathbf{w }}$, and invert both $\mathbf{G }_{\mathbf{w }}$ and $\mathbf{A }_{2,2}$. Preparing SS-$\widetilde{\text {H}}$-BLUP involves building $\mathbf{G }$, $\mathbf{A }_{2,2}$ and $\mathbf{G }_{\mathbf{w }}$, and inverting $\mathbf{G }_{\mathbf{w }}$, whereas setting up SS-T-BLUP requires building $\mathbf{M }^{\dagger }$ and $\mathbf{M }^{*}$. Furthermore, SS-$\widetilde{\text {H}}$-BLUP and SS-T-BLUP require a sparse factorisation of $\mathbf{A }^{1,1}$ to facilitate matrix-vector operations on ($\mathbf{A }^{2,2}-\mathbf{A }^{2,1}(\mathbf{A }^{1,1})^{-1}\mathbf{A }^{1,2}$) and sampling the diagonal elements of ($\mathbf{A }^{2,2}-\mathbf{A }^{2,1}(\mathbf{A }^{1,1})^{-1}\mathbf{A }^{1,2}$) if required [12]. Note that vector operations on $(\mathbf{A }^{1,1})^{-1}$ involve solving an equation for every single vector instead of doing an inversion once [11].

A widely used method when solving MME iteratively is the conditioned gradient descent method [also known as preconditioned gradient method (PCG)]. It requires the multiplication of a vector with the MME coefficient matrix once per iteration. Therefore, this method is affected by the way the inverse of $\mathbf{H }$ is presented. More specifically, during iteration the computational differences between the three approaches can be reduced to the multiplication of a vector of length $m_g$, say *z*, with a dense matrix, which is ($\mathbf{G }_{\mathbf{w }}^{-1}$ − $\mathbf{A }_{2,2}^{-1}$), or $\mathbf{G }_{\mathbf{w }}^{-1}$, or $\mathbf{M }^{*}$ $\mathbf{M }^{*^\prime}$, for SS-H-BLUP, SS-$\widetilde{\text {H}}$-BLUP and SS-T-BLUP, respectively. Furthermore, SS-$\widetilde{\text {H}}$-BLUP and SS-T-BLUP require the multiplication of *z* with the matrix ($\mathbf{A }^{2,2}-\mathbf{A }^{2,1}(\mathbf{A }^{1,1})^{-1}\mathbf{A }^{1,2}$), which involves solving ($\mathbf{A }^{1,1}$) **f**$^{*}$ = **f**, where **f**=$\mathbf{A }^{1,2}$ **z**.

Differences in peak memory requirement directly result from the size of the arrays, which must be kept in RAM simultaneously during preparation and iteration. Furthermore, for SS-H-BLUP and SS-$\widetilde{\text {H}}$-BLUP, the computational task, in which peak memory usage occurs, changes as the number of genotyped individuals exceeds the number of markers.

### Data

The SS-H-BLUP, SS-$\widetilde{\text {H}}$-BLUP and SS-T-BLUP models were applied to an Australian Angus beef cattle dataset currently used in BREEDPLAN single step genetic evaluation [13]. The dataset comprised 35 traits with 9,565,814 records and 2,621,403 individuals in the pedigree. The number of animals with genotypes was 58,705 which comprised SNP genotypes of various densities and panel manufacturers imputed to a common set of 56,009 SNPs [14]. To increase the computational load, additional 91,295 and 341,295 dummy genotypes (total dataset size of 150k and 400k genotypes, respectively) were generated in a regression-sampling approach (see next paragraph). The 400k dataset was used only for SS-T-BLUP because the other models were computationally infeasible.

Dummy genotypes for 91,295 (341,295) individuals, sampled from the pool of non-genotyped individuals, were generated by $\widetilde{\mathbf{M }}=\mathbf{A }_{*,2}\mathbf{A }_{2,2}^{-1}\mathbf{M }_{\mathbf{c }}$, where $\widetilde{\mathbf{M }}$ is a matrix of dimension 91,295 (341,295) $\times$ 56,009 of expected marker counts of the sampled non-genotyped individuals, $\mathbf{M }_c$ is a matrix of real marker counts of dimension 58,705 $\times$ 56,009, which were centred using mean allele counts estimated from the data, and $\mathbf{A }_{*,2}$ is the off-diagonal block of $\mathbf{A }$ between the sampled non-genotyped individuals and the 58,705 genotyped individuals. Outliers in $\widetilde{\mathbf{M }}$ ($<0$ and $>2$) were truncated to 0 and 2, respectively, where the proportion of outliers was lower than 1%. Subsequently, each expected marker count $\widetilde{\mathbf{M }}_{i,j}$ was translated into a dummy marker genotype by drawing two samples from a binomial distribution with parameters $p=\widetilde{\mathbf{M }}_{i,j}/2$ and $q=1-\widetilde{\mathbf{M }}_{i,j}/2$. Note that dummy genotypes that are generated this way may be affected by Mendelian inconsistency, but these were only generated for the purpose of increasing the computational load and are not part of the usual BREEDPLAN analysis.

The BREEDPLAN multi-trait model included pre-corrected phenotypes [15], a single fixed factor per trait, 27 correlated random genetic factors (including direct and maternal), 27 correlated random genetic group factors with 19 genetic groups (including direct and maternal), 3 correlated random maternal permanent environmental factors and 22 correlated random sire-by-herd interaction factors. For traits with repeated observations, repetitions were modelled as correlated traits sharing the same genetic factor. Accounting for the extensive production system and the widespread use of natural mating in large herds using groups of bulls, the pedigree and its derivatives (e.g. $\mathbf{A }$, $\mathbf{A }^{-1}$) allowed for more than one pair of parents per animal if necessary [15]. The total number of equations was 76,823,378.

For all three models matrix, $\mathbf{D }$ was set to identity, matrix $\mathbf{C }$  = $\mathbf{A }_{2,2}$, and $\lambda$ and $\gamma$ were set as 0.05 and 0.95, respectively.

### Software

The system of equations was solved with AGBU’s current large-scale linear mixed model library solver, which uses the PCG algorithm for iteratively solving linear mixed models and integrates Intel(R) MKL(R), version 2017 update 8. For research and commercial purposes, the solver is available on request. Block-diagonal and diagonal pre-conditioners were used for random and fixed factors, respectively.

Denoting the MME as **Xb** = **y**, where **X** is the coefficient matrix, **b** is the solution vector and **y** is the right hand side vector, convergence was achieved when the L2 norm of vector (**y** − **Xb**) scaled by the L2 norm of vector **y** was $\le 2.68E^{-9}$. All computationally relevant integers and all real numbers were represented in 64 bit form. All matrices and vectors required for preparation and solving were stored in random access memory (RAM). Computations for the 150k dataset were carried out on a computer with two sockets each with an Intel(R) Xeon(R) CPU E5-2697 v3 with 2.60 GHz, a total of 28 cores and 528 GB of RAM. Computations for the 400k dataset were carried out on a computer with two sockets each with an Intel(R) Xeon(R) CPU E5-2697 v4 with 2.30 GHz, a total of 36 cores and 256 GB of RAM.

## Results

**Fig. 1:**
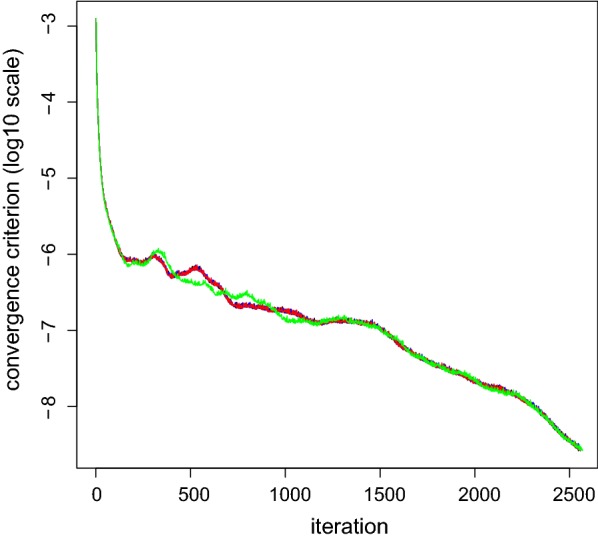
The solver convergence criterion on a log$_{10}$ scale as a function of the number of iterations for SS-H-BLUP$_{150}$ (black), SS-$\widetilde{\text {H}}$-BLUP$_{150}$ (blue), SS-T-BLUP$_{150}$ (red) and SS-T-BLUP$_{400}$ (green)

Results for the different parts of the setup and solving steps are in Table 1. SS-H-BLUP$_{150}$, SS-$\widetilde{\text {H}}$-BLUP$_{150}$, SS-T-BLUP$_{150}$ and SS-T-BLUP$_{400}$ converged in equal numbers of rounds which was $\simeq$ 2560 (see Fig. 1). The major differences between SS-H-BLUP$_{150}$, SS-$\widetilde{\text {H}}$-BLUP$_{150}$ and SS-T-BLUP$_{150}$ were the computing times for run preparation and per round of iteration.

**Table 1:** Processing time in real time seconds (hours) for various tasks and the additional memory requirement in gigabyte specific to the model when iteratively solving a SS-T-BLUP, SS-H-BLUP and SS-$\widetilde{\text {H}}$-BLUP model using a multi-trait Australian Angus BREEDPLAN dataset with 35 traits, 2.6 million animals and 77 million equations

Task	SS-H-BLUP$_{150}^{1}$	SS-$\widetilde{\text {H}}$-BLUP$_{150}$	SS-T-BLUP$_{150}$	SS-T-BLUP$_{400}^{2}$
$\mathbf{G }$	1756	1756	–	–
$\mathbf{A }_{2,2}$	250	250	–	–
$\mathbf{G }$ $^{-1}$	9150	9150	–	–
$\mathbf{A }_{2,2}$ $^{-1}$	3500	–	–	–
$\mathbf{M }^{\dagger }$ and **K**	–	–	3422	4210
$\mathbf{K }_{\mathbf{u }}$	–	–	352	320
$\mathbf{M }^{*}$	–	–	629	1170
$\mathbf{A }_{2,2}^{-1}$ $\hbox {diag}^{3}$	–	262	262	219
Preprocessing total	14,656 (4)	11,418 (3.2)	4,665 (1.3)	5,919 (1.6)
Iteration time per round	7.5	11.2	8.6	12
Total iteration time	19,123 (5.3)	28,716 (7.9)	22,134 (6.1)	30,809 (8.5)
Total evaluation time	33,779 (9.4)	40,134 (11.1)	26,799 (7.4)	36,728 (10.2)
$\approx$ $\text {RAM}^{4}$	180	180	104	216

(1) 150,000 individuals with genotypes. (2) 400,000 individuals with genotypes. (3) Sampling of diagonal elements of $\mathbf{A }_{2,2}^{-1}$ using 10,000 samples. (4) Approximated model specific memory requirement in addition to the memory requirement common to all models. SS-H-BLUP: $\mathbf{G }_{\mathbf{w }}$ and $\mathbf{A }_{2,2}$ were build explicitly and inverted. SS-$\widetilde{\text {H}}$-BLUP: $\mathbf{G }_{\mathbf{w }}$ and $\mathbf{A }_{2,2}$ were build explicitly. $\mathbf{G }_{\mathbf{w }}$ was inverted explicitly, $\mathbf{A }^{2,2}-\mathbf{A }^{2,1}(\mathbf{A }^{1,1})^{-1}\mathbf{A }^{1,2}$ was used whilst solving. SS-T-BLUP: an implicit representation of $\mathbf{G }_{\mathbf{w }}^{-1}$ and $\mathbf{A }^{2,2}-\mathbf{A }^{2,1}(\mathbf{A }^{1,1})^{-1}\mathbf{A }^{1,2}$ were used whilst solving

The preparation time for model specific parts for SS-T-BLUP$_{150}$ was 1.3 h, for SS-H-BLUP$_{150}$ 4 h and for SS-$\widetilde{\text {H}}$-BLUP$_{150}$ 3.2 h. Thus, compared to SS-T-BLUP, SS-H-BLUP needed 3 times and SS-$\widetilde{\text {H}}$-BLUP 2.5 times more real time for all necessary pre-calculations.

In terms of time per iteration, SS-H-BLUP$$_{150}$$ needed 7.5 real time seconds for a single round of the preconditioned gradient solver, followed by SS-T-BLUP$$_{150}$$ with 8.5 real time seconds. With 11.2 seconds per iteration SS-$$\widetilde{\text {H}}$$-BLUP was slowest. These differences were caused by multiplying a vector, say **y**, with matrices $${\varvec{\Psi }}^{-1}$$, $$\mathbf{H }^{-1}$$ and $$\widetilde{\mathbf{H }}^{-1}$$. This can be narrowed down further to a single matrix vector operation $${\varvec{\Delta }}$$$$\mathbf{H }_{2,2}^{-1}\mathbf{y }=(\mathbf{G }_{\mathbf{w }}^{-1}-\mathbf{A }_{2,2}^{-1})\mathbf{y }$$ in SS-H-BLUP, or one matrix vector operation $$\mathbf{G }_{\mathbf{w }}^{-1}y$$ and one solver operation **y** = ($$\mathbf{A }^{2,2}-\mathbf{A }^{2,1}(\mathbf{A }^{1,1})^{-1}\mathbf{A }^{1,2}$$)**x** in SS-$$\widetilde{\text {H}}$$-BLUP, or two matrix vector operations $$\mathbf{M }^{*}$$ $$\mathbf{M }^{*^\prime}$$ **y** and one solver operation **y** = $$\mathbf{A }^{2,2}-\mathbf{A }^{2,1}(\mathbf{A }^{1,1})^{-1}\mathbf{A }^{1,2}$$ **x** in SS-T-BLUP. In the example given here, computations of $${\varvec{\Delta }}\mathbf{H }_{2,2}^{-1}$$**y** and $$\mathbf{G }_{\mathbf{w }}^{-1}$$ **y** required $$\approx 2.25e10$$ floating point operations (FLOP), whereas $$\mathbf{M }^{*}$$ $$\mathbf{M }^{*^\prime}$$ **y** required $$\approx$$ 1.5*e*10 FLOP.

SS-T-BLUP and SS-$\widetilde{\text {H}}$-BLUP have further computational costs for solving **y** = ($\mathbf{A }^{2,2}-\mathbf{A }^{2,1}(\mathbf{A }^{1,1})^{-1}\mathbf{A }^{1,2}$)**x**, which offset the FLOP advantage of SS-T-BLUP and produce an additional overhead for SS-$\widetilde{\text {H}}$-BLUP when compared to SS-H-BLUP. For SS-$\widetilde{\text {H}}$-BLUP$_{150}$, this disadvantage is not balanced by avoiding inversion of $\mathbf{A }_{2,2}$, which results in SS-$\widetilde{\text {H}}$-BLUP having the longest total run time of all approaches. The combination of an advantage in terms of FLOPs, extra burden for operation **y** = ($\mathbf{A }^{2,2}-\mathbf{A }^{2,1}(\mathbf{A }^{1,1})^{-1}\mathbf{A }^{1,2}$)**x** and huge saving in preparation time made SS-T-BLUP the fastest of all approaches. Note that for SS-$\widetilde{\text {H}}$-BLUP the operation **y** = ($\mathbf{A }^{2,2}-\mathbf{A }^{2,1}(\mathbf{A }^{1,1})^{-1}\mathbf{A }^{1,2}$)**x** is the only overhead compared to SS-H-BLUP when iterating, and therefore allows inference regarding the increase in seconds per iteration solely attributable to the sparse representation of $\mathbf{A }_{2,2}^{-1}$.

Due to major time savings for run preparation and only a slight increase in time per iteration, SS-T-BLUP$_{150}$ needed only 80% of the total processing time required by SS-H-BLUP$_{150}$, and only 66% of SS-$\widetilde{\text {H}}$-BLUP$_{150}$. The difference in total processing time between SS-H-BLUP$_{150}$ and SS-$\widetilde{\text {H}}$-BLUP$_{150}$ was almost 2 h caused by a rapid inversion of $\mathbf{A }_{2,2}$ and fast iteration when using SS-H-BLUP$_{150}$.

Additional approximate random access memory (RAM) requirements in gibabyte due to matrices and operations that are unique to the approaches are in the last row in Table 1. For SS-H-BLUP and SS-$\widetilde{\text {H}}$-BLUP, the additional RAM requirement peaked when $\mathbf{G }$ and $\mathbf{A }_{2,2}$ or their inverse matrices were kept in RAM to calculate $\mathbf{G }_{\mathbf{w }}$ or ($\mathbf{G }_{\mathbf{w }}^{-1}$ − $\mathbf{A }_{2,2}^{-1}$), respectively. For SS-T-BLUP the additional RAM requirement peaked when operations ($\gamma ^{-1}$ $\mathbf{D }^{-1}$ + $\mathbf{M }^{^\prime}$ $\mathbf{M }^{\dagger }$) and $\mathbf{M }^{\dagger }$ = $\lambda ^{-1}$($\mathbf{A }^{2,2}-\mathbf{A }^{2,1}(\mathbf{A }^{1,1})^{-1}\mathbf{A }^{1,2}$) $\mathbf{M }$ required keeping matrix $\mathbf{M }$ and a matrix of dimension $m_m\times m_m$ in RAM simultaneously.

The last column in Table 1 shows the computing time and additional RAM requirement for SS-T-BLUP$_{400}$. Note that SS-H-BLUP models using 400k dataset were computationally infeasible.

## Discussion

SS-T-BLUP has been proposed as a single step model which can be helpful for datasets for which the number of genotyped individuals exceeds the number of markers and the $\mathbf{G }$ matrix is algebraically not invertible. These situations are becoming more common in commercial plant and livestock species where increasing numbers of individuals are genotyped with low- to medium-density SNP chips [6]. The method is enabled by reformulating the $\mathbf{H }$ matrix representation such that neither the $\mathbf{G }$ or $\mathbf{A }_{2,2}$ matrices, nor their inverse matrices need to be built or approximated.

In terms of modelling capacity SS-T-BLUP, SS-H-BLUP, and SS-$\widetilde{\text {H}}$-BLUP have drawbacks compared to SS-SNP-BLUP. The derivation of matrix ${\varvec{\Psi }}^{-1}$ is dependent on a matrix $\mathbf{C }$ with weight $\lambda$, which is usually matrix $\mathbf{A }_{2,2}$ or a diagonal matrix of random noise. This applies to matrices $\mathbf{H }^{-1}$ and $\widetilde{\mathbf{H }}^{-1}$ as well, because invertibility of $\mathbf{G }$ is never guaranteed. In addition, SS-SNP-BLUP can be reformulated such that every single genetic effect in the model can have different $\gamma$ and $\lambda$ and every single marker in the model can have a different genetic co-variance matrix. Such a situation arises when markers have different effects within a trait and different effects in different traits. The former requires $\mathbf{D }$ to be non-identity diagonal, the latter a unique matrix $\mathbf{D }$ for every single trait. In a multi-trait analysis, the genetic covariance matrix for marker *i* may then be $\sqrt{{\mathbf{D }_{i}}}\Sigma \sqrt{{\mathbf{D }_{i}}}$ where $\Sigma$ is a global genetic co-variance matrix and $\mathbf{D }_{\mathbf{i }}$ is a diagonal matrix of weights of marker *i* in the different traits. This expansion is not possible for the models applied here. However, SS-SNP-BLUP usually comes at the cost of much higher model dimensionality and slow convergence rates when solved iteratively [3]. The latter can be dealt with by using a more elaborate pre-conditioner, which is still computationally demanding [4]. To our knowledge, it has not been shown yet that the model flexibility of SS-SNP-BLUP is required for more accurate EBV.

Since all models were equivalent, it was expected that the number of iterations needed for convergence was the same. However, surprisingly there was no difference in the number of iterations for convergence when using only the 58,705 real genotypes (results not shown), 150k genotypes or 400k genotypes. A possible explanation is the way the dummy genotypes were generated. Thus, it is very likely that a dataset with 400k real genotypes may require more iterations but that the time needed for preparation and per iteration will be similar to that observed in this study.

As shown by the results, SS-T-BLUP clearly outperforms SS-H-BLUP in terms of total processing time mainly due to the huge computational cost of setting-up $\mathbf{G }$, $\mathbf{A }_{2,2}$ and inverting both. In particular, the inversion cost grows cubic with $m_g$, whereas at a constant $m_m$ the cost for generating $\mathbf{M }^{\dagger }$ grows less than linearly and the cost for $\mathbf{K }_{\mathbf{u }}$ grows proportional to $(m_m\times (m_m+1))/2\times m_g$.

## Conclusion

These results support the conclusion that SS-T-BLUP provides a feasible algorithm to calculate exact solutions for estimated breeding values when the number of genotyped individuals exceeds the number of markers. A limitation to the number of genotyped individuals is only set by the available RAM. Therefore, SS-T-BLUP allows solving single step equation systems iteratively without generating $\mathbf{G }$ or $\mathbf{A }_{2,2}$ or their inverse matrices or any approximation of these matrices.
